# Correction to: Moving beyond the classic diferencein-diferences model: a simulation study comparing statistical methods for estimating efectiveness of state-level policies

**DOI:** 10.1186/s12874-022-01522-y

**Published:** 2022-01-30

**Authors:** Beth Ann Grifn, Megan S. Schuler, Elizabeth A. Stuart, Stephen Patrick, Elizabeth McNeer, Rosanna Smart, David Powell, Bradley D. Stein, Terry L. Schell, Rosalie Liccardo Pacula

**Affiliations:** 1 RAND Corporation, 1200 South Hayes Street, Arlington, VA 22202 USA; 2 Johns Hopkins Bloomberg School of Public Health, Baltimore, MD 21205 USA; 3 Vanderbilt University Medical Center and School of Medicine, Nashville, TN 37232 USA; 4 RAND Corporation, Santa Monica, CA 90401 USA; 5 RAND Corporation, Pittsburgh, PA 15213 USA; 6 University of Southern California, Los Angeles, CA 90089 USA

**Correction to: BMC Med Res Methodol 21, 279 (2021)**

**https://doi.org/10.1186/s12874-021-01471-y**

Following publication of the original article [1], the authors noticed a typographical error on the name of one of the authors on this article. The author name “Bradley D. Stei should be Bradley D. Stein”. The original article has been updated.
