# Supplementary material for: Moving beyond the classic difference-in-differences model: a simulation study comparing statistical methods for estimating effectiveness of state-level policies
Source: BMC Med Res Methodol. 2021 Dec 13;21:279. doi: 10.1186/s12874-021-01471-y (PMC8666265; doi:10.1186/s12874-021-01471-y)
Supplement: Supplementary file 1 — Additional file 1. [file 12874_2021_1471_MOESM1_ESM.docx]

Code. We are currently finishing a codebase that will be placed on GitHub allowing for easy replication and extension of our code for running the simulations in this study. Here, we copy a sample run for one model (the unweighted linear two-way fixed effects model) over the null and assuming a 5% effect of the simulated law.

rm()

library(DataCombine)

library(MASS)

library(sandwich)

library(lmtest)

#############################################

##Functions

#############################################

#function needed for slow coding

const<-function(m)

{

v=0

if(m!=0)

{

for(i in 1:m)

{

v=v+i

}

}

return(v)

}

#function needed for slow coding

slow.acting<-function(month,length,monthly.effect)

{

top=length-1

total.times<-c(1:top) #creating length+1 spline values for the slow acting time span

#compute year 1 average effect

Fraction.year.enacted<-(13-month)/12

Average.effect.while.enacted =0.5*Fraction.year.enacted*(1/length)

Average.effect.over.year1 = Fraction.year.enacted*Average.effect.while.enacted

values.midyrs<-Average.effect.over.year1 + total.times*(1/length)

value.last.yr<-((13-month)*1+(month-1)-const(month-1)*monthly.effect)/12

values=c(Average.effect.over.year1,values.midyrs,value.last.yr)

return(values)

}

# calculate mean squared error

mse<-function(x)

{

return(mean(x^2,na.rm=T))

}

#Set p-values so denote if result statistically signifcant at alpha = 0.05 level

#0 for p>=0.05 and 1 for p<0.05

pval.bin<-function(p)

{

p[p<0.05]=1

p[p!=1]=0

return(p)

}

# Calculate correction factor for standard error

corr.factor<-function(t.stats)

{

f.stats=(t.stats)^2

f.stats=sort(f.stats)

high.cut=0.95*iters

femp95=f.stats[high.cut]

freal=qf(.95,1,Inf)

corr.factor=sqrt(femp95/freal)

return(corr.factor)

}

#formula for correcting p-values using correction factor

adj.ps<-function(regn.coeffs,ses,cf)

{

adj.ses=sqrt(ses)*cf

low95=regn.coeffs-1.96*adj.ses

high95=regn.coeffs+1.96*adj.ses

new.p=rep(0,iters)

for(i in 1:iters)

{

if(low95[i]<0&high95[i]>0)

{

new.p[i]=1

}else{

new.p[i]=0

}

}

return(new.p)

}

#type S

type.s<-function(betas,pvals,effect.direction)

{

if(length(betas[pvals<0.05])!=0)

{

if(effect.direction=="neg"){

a=length(betas[betas>0&pvals<0.05])/length(betas[pvals<0.05])

}else{

a=length(betas[betas<0&pvals<0.05])/length(betas[pvals<0.05])

}

}else{

a=0

}

return(a)

}

#correct rejection rate

test.cf<-function(regn.coeffs,ses,cf,effect.direction)

{

adj.ses=sqrt(ses)*cf

low95=regn.coeffs-1.96*adj.ses

high95=regn.coeffs+1.96*adj.ses

new.p=rep(0,iters)

for(i in 1:iters)

{

if(low95[i]<0&high95[i]>0)

{

new.p[i]=0

}else{

new.p[i]=1

}

}

#switch findings in the incorrect direction to 0's

if (effect.direction == "pos"){

new.p[new.p==1&regn.coeffs<0]=0

}else{

new.p[new.p==1&regn.coeffs>0]=0

}

return(sum(new.p)/iters) #should be ~0.05

}

#needed for performing cluster adjustment to standard errors

robust.se <- function(model, cluster){

require(sandwich)

require(lmtest)

M <- length(unique(cluster))

N <- length(cluster)

K <- model$rank

dfc <- (M/(M - 1)) * ((N - 1)/(N - K))

uj <- apply(estfun(model), 2, function(x) tapply(x, cluster, sum));

rcse.cov <- dfc * sandwich(model, meat = crossprod(uj)/N)

rcse.se <- coeftest(model, rcse.cov)

return(list(rcse.cov, rcse.se))

}

#################################

#Simulation function

#################################

# The main simulation generator and output function

run.sim = function(code.speed, effect.direction){

#creating matrix to hold 4 key regression results a

#Column 1 = estimated effect (regression coefficient)

#Column 2 = estimated variance

#Column 3 = t-statistic

#Column 4 = p-value

stats.matrix1=list(matrix(0,iters,4),matrix(0,iters,4),matrix(0,iters,4),matrix(0,iters,4))

stats.matrix1h=list(matrix(0,iters,4),matrix(0,iters,4),matrix(0,iters,4),matrix(0,iters,4))

stats.matrix1cl=list(matrix(0,iters,4),matrix(0,iters,4),matrix(0,iters,4),matrix(0,iters,4))

stats.matrix1hcl=list(matrix(0,iters,4),matrix(0,iters,4),matrix(0,iters,4),matrix(0,iters,4))

#use same seed for all runs so run on same simulated dataset for each model

set.seed(1234567)

#outer loop covers 4 different treated states sample sizes

for(j in 1:4)

{

n.trt=n.states[j]

#inner loop created the needed iters of simulated datasets

for(k in 1:iters)

{

#Create vector of state names to sample from

state.names=as.character(unique(x$STATE))

#randomly sample the exposed/treated states

z=sample(state.names,n.trt,replace=FALSE)

#randomly sample the year the law was enacted for each treated states

years.enacted=sample(c(2002:2013),n.trt,replace=TRUE)

#randomly sample the month the law was enacted for each treated state

month.enacted=sample(c(1:12),n.trt,replace=TRUE)

#create levels coding

x$levels.coding=rep(0,nrow(x))

if (code.speed == "slow") {

#Slow coding - assumes it takes 3 years for a law to become fully effective

length=3

#loops through for each treated/exposed state to create the needed slow coding levels coding

for(s in 1:n.trt)

{

month=month.enacted[s]

values=slow.acting(month,length,monthly.effect = (1/length)/12)

mark=length+1

mark2=years.enacted[s]+length

check=2016-years.enacted[s]

if(check>=length(values))

{

x$levels.coding[x$STATE==z[s]&x$YEAR>=years.enacted[s]][1:mark]=values

x$levels.coding[x$STATE==z[s]&x$YEAR>mark2]=1

}else{

hold=check+1

x$levels.coding[x$STATE==z[s]&x$YEAR>=years.enacted[s]][1:hold]=values[1:hold]

}

}

#Creating change levels coding for models for treated/exposed states

x$ch.levels.coding=rep(0,nrow(x))

for(s in 1:n.trt)

{

levels=x$levels.coding[x$STATE==z[s]]

levels.shifted=c(0,levels[-length(levels)])

x$ch.levels.coding[x$STATE==z[s]]=levels-levels.shifted

]

}else{

if (code.speed == "instant"){

#Instantaneous coding version

for(s in 1:n.trt)

{

x$levels.coding[x$STATE==z[s]&x$YEAR==years.enacted[s]]=(12-month.enacted[s]+1)/12

x$levels.coding[x$STATE==z[s]&x$YEAR>years.enacted[s]]=1

}

#Creating change levels coding

x$ch.levels.coding=rep(0,nrow(x))

for(s in 1:n.trt)

{

levels=x$levels.coding[x$STATE==z[s]]

levels.shifted=c(0,levels[-length(levels)])

x$ch.levels.coding[x$STATE==z[s]]=levels-levels.shifted

}

}

}

#GENERATING OUTCOMES

if(effect.direction !="null"){

##################

#Introduce treatment effects to state observations

##################

if (link == "linear"){

x$cr.adj=x$Crude.Rate+te*x$levels.coding

}

if (link == "log-lin"){

x$logY.adj=log(x$Crude.Rate+x$Crude.Rate*(te-1)*x$levels.coding)

x$cr.adj=exp(x$logY.adj)

}

if (link == "log"){

x$deaths.adj=x$Deaths+x$Deaths*(te-1)*x$levels.coding

x$deaths.adj=round(x$deaths.adj)

x$cr.adj=(x$deaths.adj*100000)/x$POPULATION

}

#need lags to be computed on new adjusted crude rates as potential control covariate in models

mark1=dim(x)[2]+1

x <- slide(x, Var = "cr.adj", GroupVar = "STATE", slideBy = -1)

colnames(x)[mark1] <- "lag1"

#x$lag1 = x$cr.adj.lag1

}else{

x$cr.adj=x$Crude.Rate

x$deaths.adj=x$Deaths

x$lag1 = x$crude.rate.lag1

x$logY.adj=log(x$Crude.Rate)

x$lag1 = x$cr.adj.lag1

}

#####################################################

# Insert Regression Model Here - Illustrative Example

# the formula line below can be changed to include different effects,

# including lags (use variable "lag1"), change-levels coded effects variables.

# the model line can substitute other models.

#let's test two way fixed effects WITHOUT population weights

m1=lm(cr.adj~levels.coding+as.factor(YEAR)+as.factor(STATE)+ UNEMPLOYMENTRATE,data=x)

#####################################################

#store results

stats.matrix1[[j]][k,1]= summary(m1)$coefficients[2,1] #regression coefficient

stats.matrix1[[j]][k,2]= summary(m1)$coefficients[2,2]^2 #se^2

stats.matrix1[[j]][k,3]= summary(m1)$coefficients[2,3] #t-statistics

stats.matrix1[[j]][k,4]= summary(m1)$coefficients[2,4] #p-value

#Coding for implementing adjustments to standard errors

#Huber adjustment requires library("sandwich")

cov.m1<- vcovHC(m1, type="HC0")

std.err <- sqrt(diag(cov.m1))

stats.matrix1h[[j]][k,1]= coef(m1)[2]

stats.matrix1h[[j]][k,2]= std.err[2]^2 #var

stats.matrix1h[[j]][k,3]= coef(m1)[2]/std.err[2]

stats.matrix1h[[j]][k,4] =2*pnorm(abs(coef(m1)/std.err), lower.tail=FALSE)[2]

# Arellano

cov.m1<- vcovHC(m1, type="HC1",cluster="STATE",method="arellano")

std.err <- sqrt(diag(cov.m1))

stats.matrix1hcl[[j]][k,1]= coef(m1)[2]

stats.matrix1hcl[[j]][k,2]= std.err[2]^2 #var

stats.matrix1hcl[[j]][k,3]= coef(m1)[2]/std.err[2]

stats.matrix1hcl[[j]][k,4] =2*pnorm(abs(coef(m1)/std.err), lower.tail=FALSE)[2]

#Cluster adjustment only

#Create the new variable with appropriate level names.

clustervar<-mapply(paste,"State.",x$STATE,sep="")

#Save the coefficient test output to an element in the model object

m1$coefficients<-robust.se(m1,clustervar)[[2]]

stats.matrix1cl[[j]][k,1]= m1$coefficients[2,1] #bias

stats.matrix1cl[[j]][k,2]= m1$coefficients[2,2]^2 #var

stats.matrix1cl[[j]][k,3]= m1$coefficients[2,3]

stats.matrix1cl[[j]][k,4]= m1$coefficients[2,4] #p-value

#######################################################

# remove generic lag variable

x = x[, -which(names(x) == "lag1")]

print(k)

} #ends k loop

print(j)

} #ends j loop

########################################################

#Compute Summary Statistics for runs

########################################################

if (effect.direction == "null"){

#expanding so this holds 16 rows for 4 n.trt times 4 SE models

stats1=matrix(0,16,5)

#loop through 4 sample sizes for the number of treated states

cols=c(1,2,5)

for(j in 1:4)

{

mark1=(j-1)*4+1

mark2=mark1+1

mark3=mark2+1

mark4=mark3+1

#Computes Type I Error

stats1[mark1,4]=mean(pval.bin(stats.matrix1[[j]][,4]))

stats1[mark2,4]=mean(pval.bin(stats.matrix1h[[j]][,4]))

stats1[mark3,4]=mean(pval.bin(stats.matrix1cl[[j]][,4]))

stats1[mark4,4]=mean(pval.bin(stats.matrix1hcl[[j]][,4]))

#Computes Simple Mean Summaries for the other columns

stats1[mark1,cols]=apply(stats.matrix1[[j]][,1:3],2,mean)

stats1[mark2,cols]=apply(stats.matrix1h[[j]][,1:3],2,mean)

stats1[mark3,cols]=apply(stats.matrix1cl[[j]][,1:3],2,mean)

stats1[mark4,cols]=apply(stats.matrix1hcl[[j]][,1:3],2,mean)

#Computes MSE under null

stats1[mark1,3]=mse(stats.matrix1[[j]][,1])

stats1[mark2,3]=mse(stats.matrix1h[[j]][,1])

stats1[mark3,3]=mse(stats.matrix1cl[[j]][,1])

stats1[mark4,3]=mse(stats.matrix1hcl[[j]][,1])

}

file1=paste("Summaries_Null_",code.speed,"_", model.name,".csv",sep="")

file1=paste("Summaries_Null_",code.speed,"_", model.name,".csv",sep="")

n.states.exp=c(rep(n.states[1],4),rep(n.states[2],4,),rep(n.states[3],4),rep(n.states[4],4))

se.adj=c(rep(c("none","Huber","Cluster","Huber-Cluster"),4))

stats1=as.data.frame(cbind(n.states.exp,se.adj,stats1))

names(stats1)<-c("n.trt","se.adj","RegnCoeff","AveModelSE","MSE","TypeI","Tstat")

write.table(stats1,file=file1,sep=",",row.names=FALSE)

#Compute Correction Factors

stats1.cf=rep(0,16)

#compute for each number of treated/exposed states

for(j in 1:4)

{

mark1=(j-1)*4+1

mark2=mark1+1

mark3=mark2+1

mark4=mark3+1

stats1.cf[mark1]=corr.factor(stats.matrix1[[j]][,3])

stats1.cf[mark2]=corr.factor(stats.matrix1h[[j]][,3])

stats1.cf[mark3]=corr.factor(stats.matrix1cl[[j]][,3])

stats1.cf[mark4]=corr.factor(stats.matrix1hcl[[j]][,3])

}

file2=paste("Correction_Factors_",code.speed,"_",model.name,".csv",sep="")

write.table(stats1.cf,file2,sep=",",row.names=F)

# if instead it is a positive or negative effect run...

}else{

stats1=matrix(0,16,3)

#Calculate bias

if (link=="linear"){

for(j in 1:4)

{

mark1=(j-1)*4+1

mark2=mark1+1

mark3=mark2+1

mark4=mark3+1

#bias

tot.pop=sum(as.numeric(x$POPULATION))

ave.pop.per.yr=tot.pop/length(unique(x$YEAR))

APS = ave.pop.per.yr/100000

TE = target.d

stats1[mark1,1]=mean(stats.matrix1[[j]][,1]*APS-TE)

stats1[mark2,1]=mean(stats.matrix1h[[j]][,1]*APS-TE)

stats1[mark3,1]=mean(stats.matrix1cl[[j]][,1]*APS-TE)

stats1[mark4,1]=mean(stats.matrix1hcl[[j]][,1]*APS-TE)

}

}else{

for(j in 1:4)

{

mark1=(j-1)*4+1

mark2=mark1+1

mark3=mark2+1

mark4=mark3+1

tot.deaths=sum(x$Deaths)

ave.per.yr=tot.deaths/length(unique(x$YEAR))

ADPY = ave.per.yr

TE = target.d

stats1[mark1,1]=mean((exp(stats.matrix1[[j]][,1])-1)*ADPY-TE)

stats1[mark2,1]=mean((exp(stats.matrix1h[[j]][,1])-1)*ADPY-TE)

stats1[mark3,1]=mean((exp(stats.matrix1cl[[j]][,1])-1)*ADPY-TE)

stats1[mark4,1]=mean((exp(stats.matrix1hcl[[j]][,1])-1)*ADPY-TE)

}

}

#########################

#adjusted power & adjusted type S error - requires correction Factor

file2=paste("Correction_Factors_",code.speed,"_",model.name,".csv",sep="")

cfs=read.table(file2,sep=",",h=T)

for(j in 1:4)

{

mark1=(j-1)*4+1

mark2=mark1+1

mark3=mark2+1

mark4=mark3+1

#power

stats1[mark1,2]=test.cf(stats.matrix1[[j]][,1],stats.matrix1[[j]][,2],cfs$x[mark1],effect.direction)

stats1[mark2,2]=test.cf(stats.matrix1h[[j]][,1],stats.matrix1h[[j]][,2],cfs$x[mark2],effect.direction)

stats1[mark3,2]=test.cf(stats.matrix1cl[[j]][,1],stats.matrix1cl[[j]][,2],cfs$x[mark3],effect.direction)

stats1[mark4,2]=test.cf(stats.matrix1hcl[[j]][,1],stats.matrix1hcl[[j]][,2],cfs$x[mark4],effect.direction)

#type S error

stats1[mark1,3]=type.s(stats.matrix1[[j]][,1],adj.ps(stats.matrix1[[j]][,1],stats.matrix1[[j]][,2],cfs$x[mark1]),effect.direction)

stats1[mark2,3]=type.s(stats.matrix1h[[j]][,1],adj.ps(stats.matrix1h[[j]][,1],stats.matrix1h[[j]][,2],cfs$x[mark2]),effect.direction)

stats1[mark3,3]=type.s(stats.matrix1cl[[j]][,1],adj.ps(stats.matrix1cl[[j]][,1],stats.matrix1cl[[j]][,2],cfs$x[mark3]),effect.direction)

stats1[mark4,3]=type.s(stats.matrix1hcl[[j]][,1],adj.ps(stats.matrix1hcl[[j]][,1],stats.matrix1hcl[[j]][,2],cfs$x[mark4]),effect.direction)

}

if (link=="linear"){

ave.coefficient=stats1[,1]+TE

bt=abs(te)

}else{

ave.coefficient=stats1[,1]+TE

bt=abs(log.te)

}

ll = list(stats1,ave.coefficient,bt)

file3=paste("Results_",effect.direction,"_",code.speed,"_", model.name,".Rdata",sep="")

save(ll,file=file3)

}

}

#############################################

#Step 1: Prepare the data for the simulation

#############################################

setwd("FILL IN")

load("optic_sim_data_exp.Rdata")

####################################################################################################

#Step 2. Set general simulation parameters

####################################################################################################

# number of iterations

iters = 5000

# effect coding, slow or instant

code.speed = c("instant","slow")[1] #select coding scheme

# link type

# select what type of effect modeling - linear = 1; log-linear = 2; log/count = 3

link = c("linear", "log-lin", "log")[1]

# name for current model

model.name = "Opioid_Mortality_Runs_linear_2wayfe_unwt_smES"

#Creating 4 variations in sample size that we study

#Number of exposed/treated states = 1, 5, 15, and then 30

n.states=c(1,5,15,30)

##############################################################

# Step 3. Run null models (instant and slow), and positive

# and negative models (instant and slow)

#############################################################

# cycle through simulations of the null, and positive and negative effects

for (i in c("null","pos","neg")){

if (i != "null"){

#Generate effect magnitudes

if (link=="linear"){

#SIMULATING NONZERO POSITIVE EFFECTS FOR LINEAR MODELS

#first we figured out what % change equals ~700 deaths

tot.pop=sum(as.numeric(x$POPULATION))

ave.pop.per.yr=tot.pop/length(unique(x$YEAR))

APS = ave.pop.per.yr/100000

target.d=700

TE = target.d

te=TE/APS

if (i=="neg")

{

te=-te

target.d=-target.d

}

}else{

#SIMULATING NONZERO EFFECTS FOR COUNT MODELS AND LOG(Y) MODELS

#first we figured out what % change equals ~700 deaths

tot.deaths=sum(x$Deaths)

ave.per.yr=tot.deaths/length(unique(x$YEAR))

target.d=700

percent.change=target.d/ave.per.yr

if (i=="neg"){

delta=1-percent.change

target.d=-target.d

}else{

delta=1+percent.change

}

te=delta

log.te=log(delta)

}

}

# for each null, positive, or negative effect

# cycle through simulations with instant and slow coding

for (j in c("instant","slow")){

dummy = run.sim(effect.direction=i, code.speed = j)

}

}

##############################################################

# Step 4. Organize resulting data

#############################################################

for (i in c("instant","slow")){

file3=paste("Results_","neg","_",i,"_", model.name,".Rdata",sep="")

load(paste(file3,sep=""))

ave.coefficient.neg = ll[[2]]

results.neg.bias = ll[[1]][,1]

results.neg.power = ll[[1]][,2]

results.neg.typeS = ll[[1]][,3]

file3=paste("Results_","pos","_",i,"_", model.name,".Rdata",sep="")

load(paste(file3,sep=""))

ave.coefficient.pos = ll[[2]]

results.pos.bias = ll[[1]][,1]

results.pos.power = ll[[1]][,2]

results.pos.typeS = ll[[1]][,3]

if(link=="log"){bt.count=ll[[3]]} else{bt.linear=ll[[3]]}

if(link=="log-lin"){bt.count=ll[[3]]} else{bt.linear=ll[[3]]}

#power

results.power=(results.neg.power+results.pos.power)/2

#type S

results.typeS=(results.neg.typeS+results.pos.typeS)/2

#bias

results.bias=(results.neg.bias+results.pos.bias)/2

results.magbias=(results.pos.bias-results.neg.bias)/2

n.states.exp=c(rep(n.states[1],4),rep(n.states[2],4,),rep(n.states[3],4),rep(n.states[4],4))

se.adj=c(rep(c("none","Huber","Cluster","Huber-Cluster"),4))

all.results=cbind(n.states.exp,se.adj,results.bias,results.magbias,results.typeS,results.power)

all.results<-as.data.frame(all.results)

names(all.results)<-c("n.states.exp","se.adj","results.bias","results.magbias","results.typeS","results.power")

file4 = paste("Results_NonZeroEffect_",i,"_",model.name,".csv",sep="")

write.table(all.results,file=file4,sep=",",row.names=FALSE)

}

#compile into 2 columns

file4 = paste("Results_NonZeroEffect_","slow","_",model.name,".csv",sep="")

slow.results = read.table(file4,sep=",",header=TRUE)

file4 = paste("Results_NonZeroEffect_","instant","_",model.name,".csv",sep="")

instant.results =read.table(file4,sep=",",header=TRUE)

results.power=cbind(instant.results$results.power,slow.results$results.power)

results.typeS=cbind(instant.results$results.typeS,slow.results$results.typeS)

results.bias=cbind(instant.results$results.bias,slow.results$results.bias)

results.magbias=cbind(instant.results$results.magbias,slow.results$results.magbias)

n.states.exp=c(rep(n.states[1],4),rep(n.states[2],4,),rep(n.states[3],4),rep(n.states[4],4))

se.adj=c(rep(c("none","Huber","Cluster","Huber-Cluster"),4))

all.results=cbind(n.states.exp,se.adj,results.bias,results.magbias,results.typeS,results.power)

all.results<-as.data.frame(all.results)

names(all.results) =c("n.states","se.adj","results.bias.instant","results.bias.slow","results.magbias.instant", "results.magbias.slow","results.typeS.instant","results.typeS.slow",

"results.power.instant","results.power.slow")

file4 = paste("All_Results_NonZeroEffect_",model.name,".csv",sep="")

write.table(all.results,file4,sep=",",row.names=F)
